# Efficacy of Ruiyun procedure for hemorrhoids combined simplified MilliganMorgan hemorrhoidectomy with dentate line-sparing in treating grade III/IV hemorrhoids: a retrospective study

**DOI:** 10.1186/s12893-021-01251-x

**Published:** 2021-05-20

**Authors:** Qiuxiang Yu, Congcong Zhi, Lansi Jia, Hui Li

**Affiliations:** Department of Proctology, China-Japan Friendship Hospital, No. 2 Yinghua East Street, Chaoyang District, Beijing, 100029 China

**Keywords:** Hemorrhoids, Ruiyun procedure for hemorrhoids, Simplified MilliganMorgan hemorrhoidectomy, Efficacy, Safety, Postoperative complications

## Abstract

**Background:**

Hemorrhoids are common. Hemorrhoidectomy should typically be offered to patients whose symptoms result from external hemorrhoids or combined internal and external hemorrhoids with prolapse (grades III/IV). However, none of the currently used surgical methods could be considered an ideal surgical option that is effective, safe, and painless. We hypothesized that a combination of Ruiyun procedure for hemorrhoids (RPH) and simplified MilliganMorgan hemorrhoidectomy (sMMH) will increase the safety and effectiveness of surgical treatment hemorrhoids. This study aimed to evaluate the efficacy of Ruiyun procedure for hemorrhoids combined simplifiedMilliganMorgan hemorrhoidectomy with dentate line-sparing (RPH+sMMH) to treat grade III/IV hemorrhoid.

**Methods:**

Total 452 patients with hemorrhoids of grade III/IV were retrospectively reviewed in China-Japan Friendship Hospital, 244 cases were assigned to RPH+sMMH group, and 208 cases in MMH group. The primary efficacy outcome was rate of curative at 3month after operation, and the recurrence rate within 12months post operation. Secondary efficacy outcomes included wound healing time, time required to resume normal work, constipation symptom, quality of life, and pain post operation was also evaluated. The safety outcome included postoperative complications.

**Results:**

There were no differences between the two groups in demographic characteristics. There was no statistically significant difference between the two groups in the curative rate. The recurrence rate after 12months post operation in the RPH+sMMH (3.0%) was significantly lower than the sMMH group (7.8%) (P=0.032). The wound healing time was significantly shorter in RPH+sMMH group than that in MMH group (P<0.001). The time required to resume normal work in the RPH+sMMH group was significantly shorter than MMH group (P<0.001). Compared with the MMH group, the RPH+sMMH therapy preserve better life quality and lower constipation symptom (all P<0.05). Patients who underwent RPH+sMMH had significantly less postoperative pain than MMH therapy. The total rate of patients with postoperative complications in the RPH+sMMH group (8.6%) was significant lower than the MMH group (16.3%) (P=0.012).

**Conclusion:**

RPH+sMMH may more effective in treating patients with III/IV hemorrhoids, which indicated lower recurrence rate, lower postoperative complications and pain, shorter recovery and return to normal life.

## Background

Hemorrhoids are a common anorectal complaint that is often considered as benign. Hemorrhoids seem to affect people equally between genders and typically occur during middle age, though younger patients are not uncommon. Although the exact incidence in worldwide remains unknown, a study have revealed an overall prevalence of 39% for grades I to IV hemorrhoids classified according to the international classification of hemorrhoids in the current adult population [1]. In 2006 and 2007, approximately 25,000 haemorrhoidal procedures were performed in England as hospital day-case or inpatient admissions [2], therefore, hemorrhoids have become a major medical and socioeconomic problem. Currently, the MilliganMorgan open hemorrhoidectomy (MMH) is considered as the standard surgical procedure for hemorrhoid. The disadvantage of the MMH includes prolonged wound-healing, severe postoperative pain, and affects the fine sensory function of the anus [3]. The modern surgical treatment of hemorrhoids puts forward higher requirements for the protection of anal function. The reasonable protection of the dental line area, the transitional mucosal zone and the skin of the anal canal is a basic link that must be paid attention to in the operation of hemorrhoids. Hence, Rubber band ligation (RBL), infrared photocoagulation and sclerotherapy are recommended in cases of bleeding due to grade I and II hemorrhoids [4]. However, post-operation efficacy often responded with deterioration, and less than half of patients could remain asymptomatic after 4years [5]. The simplified MilliganMorgan (sMMH) is a surgical option derived from MMH. The Ruiyun procedure for hemorrhoids (RPH) is a newly developed therapy based on the RBL and is mainly applied for hemorrhoids [6]. Studies have shown that RPH can reduce the incidence of postoperative complications and improve efficacy in mixed hemorrhoids [7].

In this study, we combined RPH with sMMH to form a new surgical method named as "RPH combined sMMH with dentate line-sparing" (RPH+sMMH). This study aimed to evaluate the efficacy and safety of RPH+sMMH therapyin treating grade III/IV hemorrhoidsand explored operation indications, contraindications, steps and key points, complications.

## Materials and methods

### Participants

A total of 452 patients with hemorrhoids of grade III/IV were retrospectively reviewed in ChinaJapan Friendship Hospital from October 2018 to October 2019. Subjects who meet the diagnostic criteria of Guidelines for Clinical Diagnosis and Treatment of Hemorrhoids according to Colorectal and Anal Surgery Group of the Chinese Medical Association (2006) were enrolled [8]. The inclusion criteria were as follows: (1) meet the criteria of III/IV degree hemorrhoids; (2) aged18years old; (3) no history of rectal or anal surgery; (4) informed written consent was obtained from patients in person or by legal guardian.

Detailed exclusion criteria comprised the following: (1) combined with other intestinal and anal diseases, such as tumor, ulcerative colorectitis, intestinal tuberculosis, Crohn's disease, anal fissure, anal fistula, perianal abscess, etc.; (2) severe cardiovascular disease (severe arrhythmia, myocardial infarction within 3months, New York Heart Association Functional Classification III and IV, systolic pressure180 or<90mmHg; (3) severe liver or kidney dysfunction; (4) allergic or scarred physique; (5) women who are pregnant or breastfeeding or during menstruation; (6) A medical history of epileptic history of major depression or major anxious, or other mental disorders.

### Study design

According to the received treatments, patients were grouped in two. The treatment group received RPH combined sMMH with dentate line-sparing (RPH+sMMH), and the control group received classic MMH.

All surgery was proceeded by one surgeon (HL) under same preoperative preparations. The patients were operated in the right lateral position under intravenous general anesthesia combined local infiltration anesthesia.

**Fig. 1:**
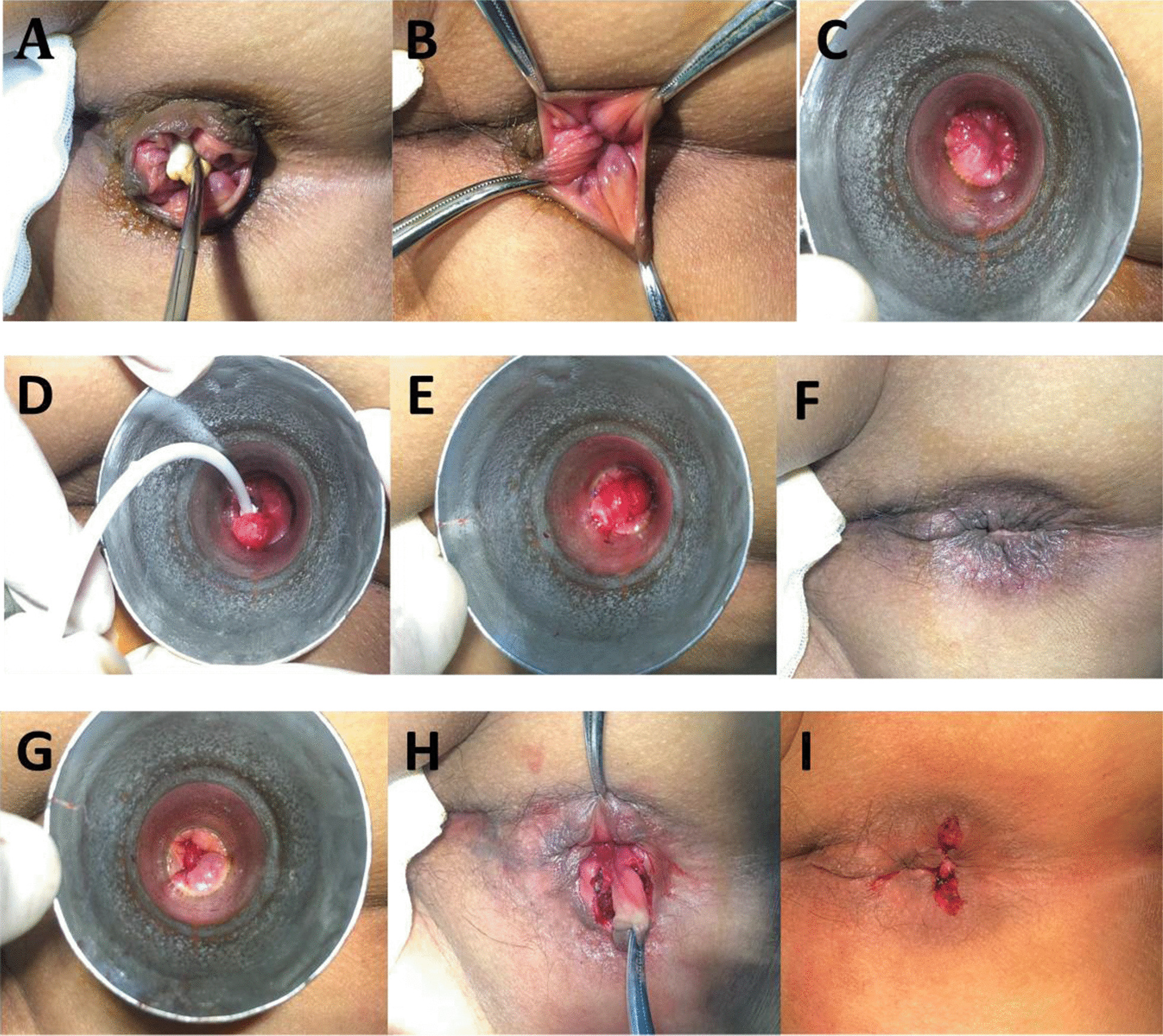
Procedure of Ruiyun procedure for hemorrhoids combined simplified MilliganMorgan hemorrhoidectomy with dentate line-sparing. **A** Circumferential prolapsed hemorrhoids with skin tags. Check the number and size of the hemorrhoids above the dentate line (**B**) and the loose mucosal part (**C**). **DF** Ruiyun procedure for hemorrhoids (RPH): suction aiming at the internal hemorrhoid or loose mucosa, turned off the negative pressure after the elastic suture ring ligated the internal hemorrhoid or loose mucosal at0.08 to0.1MPa with the elastic line fully tighten around the hemorrhoids or loose mucosal (**D**), and then remove the bandage (**E**).the prolapse has been significantly improved (**F**). **GI** Simplified MilliganMorgan procedure (sMMH): check the number and size of the hemorrhoids below the dentate line with a horn-shaped anoscope (**G**). A radial fusiform incision was made from the lower edge of the external hemorrhoids to the dentinal line, then striped the subcutaneous tissue and vein cluster, and the inverted "v" shape was formed near the dental line (**H**). Clamping the base, then ligate and remove the hemorrhoids (**I**)

The surgery procedure of RPH+sMMH for the treatment group was as follows (Fig.1): (1) Iodophor disinfects the intestinal cavity, exposing the anal canal and rectum with a horn-shaped anoscope (C); (2) carefully check the number and size of the hemorrhoids above the dentate line and the loose mucosal part (BC); (3) after confirmation of ligation point, suction aiming at the internal hemorrhoid or loose mucosa was introduced; (4) hemorrhoids were sucked into the ligation tube (try to protect the 0.5cm tissue above the dentate line), while keeping certain distance between suction barrel and intestinal wall to avoid fistula; (5) the ratchet wheel was rotated to release the elastic suture ring to ligate the internal hemorrhoid or loose mucosal at0.08 to0.1MPa with the elastic line fully tighten around the hemorrhoids (D), and then turned off the negative pressure and remove the bandage (E); (6) avoid multiple ligation points on the same level, and retain 0.5cm to 1cm mucosal bridge between the ligation points to avoid intestinal stenosis. In the process of banding, a routine intravaginal examination with a finger should be conducted to avoid torsion or pocket shape.

After the ligation was completed, the prolapse has been significantly improved (F) simplified external stripping and internal ligation was used to remove the remaining incompletely retracted hemorrhoids (G) by using curved forceps to clamp and ligate with silk thread. A radial fusiform incision was made from the lower edge of the external hemorrhoids to the dentinal line (H), then striped the subcutaneous tissue and vein cluster, and the inverted "v" shape was formed near the dental line. If the dental line was obviously raised, strip the subcutaneous tissue to 0.5cm above the dentinal line to maintain its integrity. Clamping the base, then ligate and remove the hemorrhoids (I).

The control group was assigned to receive classic MMH method, the procedures followed the criteria exposed by Milligan and Morgan [9].

Both groups were given intravenous antibiotics and analgesics for 2days. Patients were allowed enteral nutrition right after operation, high fiber diet advised and hip bath after bowel movements postoperatively.

### Outcomes

#### Primary efficacy outcome

The primary efficacy outcome was rate of curative at 3month after operation, which was evaluated according to Traditional Chinese Medicine Syndrome Diagnosis and Efficacy Standards. Clinical efficacy was classified as curative (clinical symptoms, signs, and hemorrhoids disappeared), markedly effective (clinical symptoms and signs disappeared, and hemorrhoids significantly shrunk), effective (clinical symptoms and signs improved, hemorrhoids shrunk), and ineffective (no improvement in clinical symptoms and signs, and no change in hemorrhoids) [10].

Another primary efficacy outcome was recurrence rate, the rate of recurrence was defined as symptoms and signs reappearing within 12months post operation. Recurrence was based on the findings of the patients complaint and surgeons examination.

#### Secondary efficacy outcome

The secondary efficacy outcome included: (1) wound healing time; (2) time required to resume normal work; (3) evaluation of the patient assessment of constipation symptom (PAC-SYM) [11] and patient assessment of constipation quality of life questionnaire (PAC-QOL) [12] in 3month. The PAC-SYM included 12 items, and assigned to 3 subscales: stool symptoms, rectal symptoms, and abdominal symptoms, and the range of the scores was 048, higher score means severe constipation. PAC-QOL is a brief and comprehensive assessment of the burden of constipation on patients daily functioning and well-being, 28 items was included, and the range of scores was 0 to 96, and lower scores indicated better quality of life; (4) postoperative pain: the visual analog scale for pain (VAS Pain) was used to evaluate the postoperative pain on the 1st and 10th days after surgery (010 point, higher score means severe pain).

### Safety measurement

The safety assessment included: (1) physical examination and vital signs; (2) postoperative complications, postoperative hemorrhage (blood loss800mL or20% of whole blood volume with or without systemic symptoms of blood loss, even shock), urinary retention, anal incontinence, anorectal stenosis; and (4) documentation of any adverse events that occurred during the treatment period, including the severity, time of onset, duration, treatment, and relationship to the operation.

Postoperative follow-up: data included patients characteristics and clinical history, the 1st day and10th day post operation follow up was performed an outpatient clinical examination, 3months and 12months post operation through the mobile phone software.

This study was conducted in accordance with the principles of the Declaration of Helsinki. Written informed consent was obtained from each patient who agreed to participate in this study. The study was approved by ethics committee of ChinaJapan Hospital (No. 2019-30-K24).

### Statistical analysis

The statistical analyses were conducted using SPSS 19.0. Average continuous variables with standard deviation were calculated when data are normally distributed, and the counting categorical variables were shown in percentage. The demographics between two groups were compared. ANOVA was used for testing the differences of continuous variables when data are normally distributed and equal variances assumed, and nonparametric test was used if not. The chi-squared test or Fisher exact test was used for categorical variables. The analyses of the efficacy measures were based on the observed case population. All statistical tests were two-sided, and a P<0.05 was considered statistically significant.

## Results

A total of 452 patients with mix hemorrhoids were eligible for inclusion. 244 cases were assigned to treatment group, and 208 cases in control group (Table 1). No differences were found between the two groups in gender, age, and gradeof the hemorrhoids (all P>0.05). There was no difference between the two groups regarding to the PAC-QOL and PAC-SYM (all P>0.05).

**Table 1:** Demographic characteristics of hemorrhoids patients at baseline visit

	RPH+sMMH (n=244)	MMH (n=208)	x^2^/t	P
Gender (male/female)	121/123	106/102	0.084	0.771
Age (years)	43.612.3	42.711.9	0.787	0.432
Grade of mix hemorrhoids (III/IV)	171/73	144/64	0.039	0.844
PAC-QOL	56.216.7	56.515.9	0.189	0.850
PAC-SYM	11.36.4	10.96.3	0.667	0.505
Stool symptoms	2.51.63	2.31.27	1.436	0.152
Rectal symptoms	7.74.30	7.54.53	0.489	0.631
Abdominal symptoms	2.11.63	2.01.49	0.676	0.499

*PAC-SYM* patient assessment of constipation symptom; *PAC-QOL* patient assessment of constipation quality of life questionnaire; *RPH* Ruiyun procedure for hemorrhoids; *sMMH* simplified MilliganMorgan procedure

### Primary efficacy measurement

There was no statistically difference between the two groups in the curative rate (P=0.086) (Table 2). The recurrence rate was 7 (3.0%) in the RPH+sMMH group, and the recurrence rate was 15 (7.8%) in the MMH group, there was a significant difference in recurrence rate after 12months post operation (P=0.032).

**Table 2:** Comparison of efficacy measurement between two groups

Items	RPH+sMMH	MMH	x^2^/F	P
	n=244	n=208		
Recurrence rate 12month post operation, n (%)	7 (3.0%)	15 (7.8%)	4.573	0.032
Clinical efficacy 3month post operation				
Curative, n (%)	232 (95.1%)	197 (94.5%)	0.384	0.826
Markedly effective, n (%)	9 (3.7%)	7 (3.4%)		
Effective, n (%)	3 (1.2%)	4 (1.9%)		
Ineffective, n (%)	0	0		
Wound healing time (days)	21.31.7	32.22.1	60.965	0.000
Time required to resume normal work (days)	6.51.5	15.23.3	36.951	0.000
PAC-QOL and PAC-SYM 3month post operation				
PAC-QOL	47.815.7	50.914.2	2.185	0.029
PAC-SYM	5.65.1	6.75.3	2.244	0.025
Stool symptoms	1.11.42	1.31.33	1.536	0.125
Rectal symptoms	4.53.58	5.23.1	2.205	0.028
Abdominal symptoms	1.21.0	1. 31.1	1.001	0.312
Pain not related to bowel movements post operation				
1th day	5.32.18	5.71.65	2.169	0.030
10th day	3.32.29	3.52.57	0.874	0.382
Pain during bowel movements post operation				
1th day	7.12.37	7.51.63	2.054	0.040
10th day	5.62.31	6.52.41	4.064	0.000

*RPH* Ruiyun procedure for hemorrhoids; *sMMH* Simplified MilliganMorgan procedure, *PAC-SYM* patient assessment of constipation symptom; *PAC-QOL* patient assessment of constipation quality of life questionnaire

### Secondary efficacy measurement

The wound healing time was significantly shorter in RPH+sMMH group than that in MMH group (P<0.001). The time required to resume normal work in the RPH+sMMH group was significantly shorter than MMH group (P<0.001) (Table 2).

The mean PAC-QOL in the RPH+sMMH therapy group was significantly lower than the MMH group after 3month post operation (P=0.029) (Table2), which indicated that RPH+sMMH therapy preserve better life quality. PAC-SYM scores at 3month post operation also showed significant difference between the two treatment groups (P=0.025). The rectal symptoms subscales of the PAC-SYM in the RPH+sMMH therapy was significant lower than the MMH group. There was no significant difference on the stool symptoms and abdominal symptoms between the two groups.

Patients who underwent RPH+sMMH had significantly less postoperative pain (both pain not related to bowel movements and pain during bowel movements) at the 1st day post operation than MMH therapy (all P<0.05). At the 10th day, the score of pain during bowel movements in the RPH+sMMH therapy group was significantly lower than the MMH therapy group (P<0.001), however, there was no difference on the score of pain not related to bowel movements between two groups 10th day post operation (P=0.382) (Table 2).

### Safety measurement

A comparison of incidence of complications showed in the Table 3. The total rate of patients with postoperative complications in the RPH+sMMH group (8.6%) was significant lower than the MMH group (16.3%) (P=0.012). Hemorrhage, anorectal stenosis and prolonged healing in the group that received RPH+sMMH therapy was significantly lower than in the groups that received MMH (all P<0.05); there was no difference on the urinary retention between the two groups (P=0.412) (Table 3). Seventeen patients had postoperative anal bleeding, 12 of whom required surgical hemostasis. Nineteen patients experienced acute urinary retention requiring catheterization, which was removed one to two days later. Four patients had anorectal stenosis, which was resolved after the use of anal dilator one month later. Fifteen patients had delayed wound healing, but this was resolved 2months after surgery.

**Table 3:** Comparison of postoperative complications between two groups

	RPH+sMMH	MMH	x^2^	P
	(n=244)	(n=208)		
Total n (%)	21 (8.6%)	34 (16.3%)	6.293	0.012
Hemorrhage	5 (2.0%)	12 (5.8%)	4.293	0.038
Urinary retention	12 (4.9%)	7 (3.4%)	0.672	0.412
Anorectal stenosis	0	4 (1.9%)	4.734	0.030
Delayed wound healing	4 (1.6%)	11 (5.3%)	4.660	0.031

*RPH* Ruiyun procedure for hemorrhoids; *sMMH* simplified MilliganMorgan procedure

## Discussion

Over time, conventional excisional hemorrhoid therapies have been proven to be a rather robust as a long-term solution for hemorrhoids. Many treatment options have been proposed and applied for different stages of hemorrhoids. Since MilliganMorgan established the external stripping and internal ligation in 1937, it has become the "gold standard" for the treatment of hemorrhoids for its reliable curative effect. Nevertheless, limitations also exists such as obvious postoperative pain, prolonged healing time, longer hospitalization and extended time of resumed normal work [13, 14]. In 1956, Laisdell first applied rubber band ligation (RBL) to hemorrhoids. Then, this procedure is preferred in European and American countries for its simple operation, lower pain and fewer complications. Based on RBL, our group improve the material of the rubber band and optimize surgical instruments formed RPH. However, RPH therapy is less effective for mixed hemorrhoids with severe external hemorrhoids [15]. The simplified MMH can make up for the shortcomings of RPH surgery in anal cushion suspension and raising up of the anal cushion. Also, simplified MMH can reduce the damage of MMH to anal cushions, protect dentate line and reduce the incidence of anorectal complications. Thus, the simplified MMH is only used on residual internal hemorrhoids, shrunken hemorrhoids, or incompletely retracted external hemorrhoids that occupy the anal canal after RPH surgery.

The core operation procedure of RPH was: (1) the banding can be divided into two levels. The upper layer was the upper pole of the internal hemorrhoids, and the lower layer was the internal hemorrhoids themselves, banding should avoid the dentinal line, as far as possible 0.5cm above the dentinal line; (2) the distance between ligation points on the same level should greater than 1cm. When ligating more than 3 points, a routine digital rectal examination should be conducted to avoid intestinal stenosis; (3) the larger hemorrhoids should be ligated first. If the smaller hemorrhoids are not suitable for ligation, they should be clamped with curved forceps and then ligated with silk thread. For hemorrhoids that have shrunk or incompletely retracted after RPH, sMMH was additionally performed, that is, the internal hemorrhoids were partially ligated with curved forceps, and the external hemorrhoids were partially excised. In order to protect the anal cushion, retain defecation receptors, and maintain the fine bowel control function of the anorectum, classic external resection and internal ligation are not performed.

In this study, no statistically significant difference between the two groups in the curative rate was found. But the recurrence rate in the RPH+sMMH group was significantly lower than the MMH group after 12months post operation. The relatively lower rate of recurrence in the RPH+sMMH may due to the ligation of loose mucosa on hemorrhoids, suspension and raising up of the anal cushion, and sMMH improve outlet obstruction at the same time.

In addition, we combined RPH and simplified MMH surgery to treatment III/IV degree hemorrhoid, which showed lower postoperative complications, less postoperative pain, and quicker recovery and return to normal social and working life. Besides, patients who received RPH+sMMH therapy have showed benefits on the quality of daily life and constipation symptoms, patients who received RPH+sMMH therapy, showed higher quality of life, and lighter degree of constipation. The result of our study is consistent with other controlled trials. A randomized clinical trial have evaluated the efficacy and safety of RPH+sMMH method for the treatment of III/IV degree hemorrhoids, and the results indicated the RPH+sMMH group had the highest recovery rate (97.81%) at 6months post operation, and the number of patients with postoperative hemorrhage and uroschesis was significantly lower in the group that received RPH+sMMH therapy [7].

The proportion of complications in RPH+sMMH group was significantly lower than the MMH group, hemorrhage, anorectal stenosis and prolonged healing in the group that received RPH+sMMH therapy was significantly lower than in the groups that received MMH. The possible explanation was the anal cushion was suspended and raised during the RPH therapy, and the blood flow of the hemorrhoidal artery was blocked, the hemorrhoidal tissues could be tied tightly using the suture threads with elastic loops to minimize the area of the wound. And the suture threads with elastic loops can avoid aging, fatigue and deterioration. So postoperative bleeding was reduced, combined with the sMMH therapy to remove the external hemorrhoids, and finally achieved the purpose of reducing the wound surface and protecting the anal cushion. Postoperative pain in the treatment group was less and the occurrence of urinary retention was reduced. And the results indicated that RPH+sMMH were well tolerant.

There are some limitations, however. First, the participants enrolled were under clinical observation, selection bias is the main concern of this retrospective analysis; secondly, the study sample was relatively small; finally, the 10th day post operation follow up was performed an outpatient clinical examination, but 3months and 12months postoperative follow-up were conducted through the mobile phone software, the recurrence rate within 12months post operation was assessed through pictures and complaint of patients, so the recurrence rate maybe not very precise. A prospective randomized controlled trial is needed to confirm the findings in this retrospective analysis of real world data.

## Conclusion

In conclusion, compared with MMH, RPH+sMMH may more effective in treating patients with stage III/IV hemorrhoids, RPH+sMMH may indicated lower rate of recurrence after 1 year's follow-up, lower postoperative complications, less postoperative pain, and shorter recovery and return to normal social and working life. Combined RPHwithsMMH therapy may bring additional symptomatic benefit for patients with III/IV degree hemorrhoids.

## Data Availability

Data are however available from the correspondence author upon reasonable request.

## Abbreviations

MMH: MilliganMorgan hemorrhoidectomy
PAC-SYM: Patient assessment of constipation symptom
PAC-QOL: Patient assessment of constipation quality of life questionnaire
RBL: Rubber band ligation
RPH: Ruiyun procedure for hemorrhoids
sMMH: Simplified MilliganMorgan hemorrhoidectomy
VAS: Visual analog scale for pain

## References

[1] Riss S, Weiser FA, Schwameis K, Riss T, Mittlbck M, Steiner G, Stift A (2012). The prevalence of hemorrhoids in adults. Int J Colorectal Dis.

[2] Watson AJ, Bruhn H, MacLeod K, McDonald A, McPherson G, Kilonzo M, Norrie J, Loudon MA, McCormack K, Buckley B, Brown S, Curran F, Jayne D, Rajagopal R, Cook JA (2014). A pragmatic, multicentre, randomised controlled trial comparing stapled haemorrhoidopexy to traditional excisional surgery for haemorrhoidal disease (eTHoS): study protocol for a randomised controlled trial. Trials.

[3] Kim JS, Vashist YK, Thieltges S, Zehler O, Gawad KA, Yekebas EF, Izbicki JR, Kutup A (2013). Stapled hemorrhoidopexy versus MilliganMorgan hemorrhoidectomy in circumferential third-degree hemorrhoids: long-term results of a randomized controlled trial. J Gastrointest Surg.

[4] Higuero T, Abramowitz L, Castinel A, Fathallah N, Hemery P, Laclotte Duhoux C, Pigot F, Pillant-Le Moult H, Senjoux A, Siproudhis L, Staumont G, Suduca JM, Vinson-Bonnet B (2016). Guidelines for the treatment of hemorrhoids (short report). J Visc Surg.

[5] Kanellos I, Goulimaris I, Christoforidis E, Kelpis T, Betsis D (2003). A comparison of the simultaneous application of sclerotherapy and rubber band ligation, with sclerotherapy and rubber band ligation applied separately, for the treatment of haemorrhoids: a prospective randomized trial. Colorectal Dis.

[6] Wei G, Hua X, Zhao Y (2014). Clinical study of Ruiyun procedure for hemorrhoids combined with Xiaozhiling injections in treatment of hemorrhoids complicated with human immunodeficiency virus infection. Zhonghua Wei Chang Wai Ke Za Zhi.

[7] He YH, Tang ZJ, Xu XT, Huang DQ, Zhang LS, Tang QZ, Fan ZM, Zou XJ, Zou GJ, Zhang CY, Hu F, Xie B, Li YH, Tong Y, Liu HC, Li K, Luo YL, Liu F, Situ GW, Liu ZL (2017). A randomized multicenter clinical trial of RPH with the simplified MilliganMorgan hemorrhoidectomy in the treatment of mixed hemorrhoids. Surg Innov.

[8] Chinese Society of Traditional Chinese Medicine. Guidelines for diagnosis and treatment of common disease of coloproctology in Traditional Chinese medicine. Beijing: China press of Traditional Chinese Medicine, 2012:1 (**in Chinese**)

[9] Milligan ET, Morgan CN, Jones LE, Officer R (1937). Surgical anatomy of the anal canal and operative treatment of haemorrhoids. Lancet.

[10] National administration of traditional Chinese medicine (2012). Standards for Diagnosis and Efficacy of TCM Diseases and Syndrome.

[11] Frank L, Kleinman L, Farup C, Taylor L, Miner P (1999). Psychometric validation of a constipation symptom assessment questionnaire. Scand J Gastroenterol.

[12] Marquis P, De La Loge C, Dubois D, McDermott A, Chassany O (2005). Development and validation of the Patient Assessment of Constipation Quality of Life questionnaire. Scand J Gastroenterol.

[13] Hollingshead JR, Phillips RK (2016). Haemorrhoids: modern diagnosis and treatment. Postgrad Med J.

[14] Murie JA, Sim AJ, Mackenzie I (1982). Rubber band ligation versus haemorrhoidectomy for prolapsing haemorrhoids: a long term prospective clinical trial. Br J Surg.

[15] Ali SA, Mohammad AT, Jarwar M, Imran J, Siddique AJ, Dalwani AG (2010). Outcome of the rubber band ligation with Milligan Morgan haemorrhoidectomy. J Ayub Med Coll Abbottabad.

